# Interaction between mesenchymal stem cells and myoblasts in the context of facioscapulohumeral muscular dystrophy contributes to the disease phenotype

**DOI:** 10.1002/jcp.30789

**Published:** 2022-05-27

**Authors:** Ekaterina Kiseleva, Olesya Serbina, Anna Karpukhina, Vincent Mouly, Yegor S. Vassetzky

**Affiliations:** ^1^ N. K. Koltzov Institute of Developmental Biology, RAS Moscow Russia; ^2^ Univeristy Paris‐Saclay, UMR 9018, CNRS Institut Gustave‐Roussy Villejuif France; ^3^ Sorbonne Universités, UMR‐S 974 Center for Research in Myology Paris France

**Keywords:** CXCL12, CXCR4, differentiation, fibrosis, FSHD, migration, MSCs, myoblasts

## Abstract

Facioscapulohumeral muscular dystrophy (FSHD) is a genetic disease associated with ectopic expression of the *DUX4* gene in skeletal muscle. Muscle degeneration in FSHD is accompanied by muscle tissue replacement with fat and connective tissue. Expression of *DUX4* in myoblasts stimulates mesenchymal stem cells (MSC) migration via the CXCR4‐CXCL12 axis. MSCs participate in adipose and connective tissue formation and can contribute to fibrosis. Here we studied the interaction between myoblasts and MSCs and the consequences of this interaction in the FSHD context. We used cell motility assays and coculture of MSCs with myoblasts to study their mutual effects on cell migration, differentiation, proliferation, and extracellular matrix formation. The growth medium conditioned by FSHD myoblasts stimulated MSCs migration 1.6‐fold (*p* < 0.04) compared to nonconditioned medium. Blocking the CXCL12‐CXCR4 axis with the CXCR4 inhibitor (AMD3100) or neutralizing antibodies to CXCL12 abolished this effect. FSHD myoblasts stimulated MSC proliferation 1.5−2 times (*p* < 0.05) compared to control myoblasts, while the presence of MSCs impaired myoblast differentiation. Under inflammatory conditions, medium conditioned by FSHD myoblasts stimulated collagen secretion by MSCs 2.2‐fold as compared to the nonconditioned medium, *p* < 0.03. FSHD myoblasts attract MSCs via the CXCL12‐CXCR4 axis, stimulate MSC proliferation and collagen secretion by MSCs. Interaction between MSCs and FSHD myoblasts accounts for several important aspects of FSHD pathophysiology. The CXCL12‐CXCR4 axis may serve as a potential target to improve the state of the diseased muscles.

## 1 BACKGROUND

Facioscapulohumeral muscular dystrophy (FSHD) is an autosomal dominant disorder with an overall incidence in Europe between 1:8000−20,000 (Deenen et al., 2014). FSHD is associated with an aberrant expression of the DUX4 gene, encoding for a transcription factor involved in early embryogenesis (reviewed in Karpukhina & Vassetzky, 2020). *DUX4* is heavily silenced in adult tissues and its aberrant expression in adult skeletal muscle is believed to cause muscle dysfunction (Lemmers et al., 2010; Snider et al., 2010) via a number of factors, including oxidative stress (Bosnakovski et al., 2008; Gatica & Rosa, 2016), DNA damage (Dmitriev, Bou Saada et al., 2016), and myogenesis inhibition (Gatica & Rosa, 2016; Knopp et al., 2016).

FSHD is characterized by progressive muscle weakness (Hamel & Tawil, 2018) accompanied by muscle replacement with fat and collagen (Dahlqvist et al., 2019; DeSimone et al., 2020; Stadler et al., 2011). Muscles of mice with low‐level *DUX4* expression exhibit an excess of fibro‐adipogenic progenitors, inflammation, and fibrosis (Bosnakovski et al., 2017). Immunohistochemical and magnetic resonance imaging (MRI) studies indicate an inflammatory process with lymphocyte invasion in affected FSHD muscles (Arahata et al., 1995; Frisullo et al., 2011; Tasca et al., 2012) and inflammatory conditions potentially favor muscle tissue replacement in FSHD (Dahlqvist et al., 2019).

We have previously shown that transient expression of *DUX4* in human myoblasts upregulated the expression of *CXCL12* (C‐X‐C motif ligand 12) chemokine and its receptor *CXCR4* (C‐X‐C motif receptor 4) and induced mesenchymal stem cells (MSCs) migration toward *DUX4*‐expressing myoblasts (Dmitriev, Kiseleva et al., 2016). MSCs can differentiate into adipocytes and fibroblasts (Liu et al., 2009) and are the major contributors to fibrosis in different tissues (reviewed in El Agha et al., 2017). Thus, abnormal MSC migration and accumulation in affected muscles may lead to fibrosis rather than repair of damaged muscle fibers in the FSHD context. This process may be stimulated by the inflammation characteristic for FSHD muscles.

Here, we have studied the interaction between myoblasts and MSC in the FSHD in vitro model. We demonstrated that FSHD myoblasts attracted MSCs via the CXCL12‐CXCR4 axis and stimulated MSCs proliferation. Under inflammatory conditions, MSCs conditioned by FSHD myoblasts increased collagen secretion and the presence of MSCs in the myoblast cell culture impaired myotube formation.

## 2 MATERIALS AND METHODS

### 2.1 Standard protocol approvals, registrations, and patient consents

The human muscle samples originated from the surgical waste after operations performed for medical reasons. The ethics board determined that their approval was not required in this case according to the National Legislation. Written informed consent was obtained from all patients.

### 2.2 Cell cultures

Human immortalized myoblasts (IM) of healthy and FSHD subjects were obtained from the Institute of Myology, Paris. Human primary myoblasts (PM) isolated from skeletal muscle biopsies of healthy and FSHD subjects (Barro et al., 2010) were a kind gift of Dalila Laoudj‐Chenivesse. Human MSCs from adipose tissue and TaqRFP‐MSCs were obtained from the Cell Culture Collection of IDB RAS.

Myoblasts were cultured in the medium composed of four parts of high‐glucose DMEM (Paneco) and one part of Medium 199 (Paneco), supplemented with 15% FBS (HyClone), 1Х Glutamax (Gibco), 1Х Penicillin−Streptomycin (Gibco), 10 mg/L human recombinant bFGF (Gibco), and 0.1 µM dexamethasone (Sigma). MSCs were cultured in the medium DMEM/F12 (Paneco), supplemented with 10% FBS, 1Х Glutamax, 1Х Penicillin−Streptomycin, and 1Х ITS (Gibco). MSCs were passaged using 0.05% trypsin‐EDTA (Gibco) at the 90% monolayer, myoblasts were passaged at a cell confluence not exceeding 50% to avoid cellular senescence and spontaneous differentiation.

### 2.3 Myogenic differentiation

Myoblasts were plated in high density (100% monolayer). After 24 h the growth media was replaced with myogenic induction medium: high‐glucose DMEM, 2% horse serum (Paneco), 1X Glutamax, and 1Х Penicillin−Streptomycin. When modeling inflammatory conditions, 20 ng/ml TNF‐α (Sigma) was added on Day 1 or Day 4 of differentiation. During differentiation, the medium was not changed. On the 5th day of differentiation, myoblast or cocultures were stained with May−Grunwald Giemsa dye as previously described (Velica & Bunce, 2011). Briefly, the cells were washed with PBS (Paneco), fixed with 100% methanol at +4°C for 5−10 min and air‐dried. Then the cells were incubated in May−Grunwald's solution (eosin‐methylene blue) (1:3 dilution in 1 mM PBS pH 5,6) (MiniMed) for 20 min and Giemsa solution (1:20 dilution in 1 mM PBS pH 5,6) (Paneco) for 40 min and washed with distilled water. The specimens were observed using an inverted microscope Olympus IX51 with Olympus DP70 camera. At least 10 fields of view were captured for each specimen and the images were analyzed in ImageJ. The diameter of the myotubes and the fusion index (FI) were assessed. The FI was defined as the number of nuclei inside the myotubes divided by the total number of nuclei × 100%.

### 2.4 Conditioning media by myoblasts

The growth media was washed out with serum free DMEM 24 h after myoblasts seeding, and replaced with high‐glucose DMEM, supplemented with 2% FBS, 1Х Glutamax, and 1Х Penicillin−Streptomycin. After 48 h, the conditioned medium was collected, filtered through a 0.2 µm filter and frozen at −20°C.

### 2.5 Coculture the myoblasts and MSCs

To study the effect of MSCs on myogenic differentiation and potential fusion between MSCs and myoblasts upon myotube formation, we used MSCs or TaqRFP‐expressing MSCs for MSC visualization in coculture with myoblasts. Myoblasts and MSCs/RFP‐MSCs were cocultured in various proportions, and the myogenic differentiation was induced during 5 days. After that, the cultures were fixed and stained for the MF20 marker, which only stains myotubes, myogenin or with May−Grunwald's Giemsa for FI counting.

### 2.6 Immunofluorescence staining

Cells were washed with PBS, fixed with 4% PFA for 5−10 min at +4°C and incubated in a blocking solution (0.1% triton X‐100 [Sigma‐Aldrich], 4% FBS diluted in PBS) for 30 min. Primary antibodies (Supporting Information: Table 1) diluted in a blocking solution were applied overnight at +4°C in a humid chamber. Then the cells were washed with PBS and the secondary antibodies (Supporting Information: Table 2) (diluted in blocking solution at a ratio 1:1000) were applied for 1 h at room temperature. Cell nuclei were stained with DAPI (Sigma) or PI (Biotium). The specimens were studied using an inverted fluorescence microscope Olympus IX73 equipped with Olympus DP camera. At least 10 fields of view were captured for each specimen and the images were analyzed in ImageJ (Schneider et al., 2012). FI (described above) and the percentage of myogenin‐positive nuclei were assessed.

### 2.7 Flow cytometry

The cells were detached using 0.05% trypsin‐EDTA, washed in PBS (CF 10 min 300 g), fixed with Cytofix (BD Biosciences) for 20 min at +4°C and washed again. Then the cells were resuspended in the primary antibody solution (anti‐Ki‐67 or anti‐CXCL12) (Supporting Information: Table 1) and incubated overnight at +4°C. The cells were then washed with PBS, resuspended in the secondary antibody solution (diluted in blocking solution at a ratio of 1:1000) and incubated for 1 h in the dark at room temperature. After staining, the samples were washed with PBS and analyzed using Attune NxT flow cytometer. To control for the nonspecific secondary antibodies binding, the samples nonstained with the primary antibodies were also analyzed.

### 2.8 Collagen assay

The MSCs were seeded in six wells plates (5 × 10^5^ cells); after 24 h the growth media was replaced with 5 ml of a medium containing 2% FBS, or a mix of 2% FBS medium with myoblasts‐conditioned medium in the ratio 1:1, or 2% FBS medium supplemented with 20 ng/ml TNF‐α. Collagen content was analyzed after 5 days of cultivation using the Soluble Collagen Assay Kit (QuickZyme Biosciences). In short, the medium was removed, the cells were washed with PBS, then the 0.5 M acetic acid was added and incubated overnight at +4°C on a rotating platform. Cell extracts were removed using a scraper, then centrifuged for 10 min at 3000*g*, +4°C. Сulture medium was collected and centrifuged for 10 min at 1500*g*, +4°C to remove cell debris. To quantify the collagen concentration, the bound Sirius Red dye was extracted and the optical density of the eluates was measured by plate photometer (Synergy H1, BioTek) at *λ* = 540 nm (630 nm cutoff filter).

### 2.9 Migration assays

Real‐time MSCs migration was investigated using a xCELLigence DP real‐time cell analyzer using a CIM plate for migration. The assay was performed for 25 h and the real‐time cell index measurements were continuously recorded. Cell index is a unitless parameter which reflects the impedance changes happening as the cells migrate through the membrane from the upper to the lower chamber and adhere to the electronic sensors on the underside of the membrane. Increase in the Cell Index correlates with the increasing number of migrated cells. For each well, the delta cell index was calculated as the cell index at a given time point plus a delta value. The delta value is a constant value for each well and is the difference between a reference delta cell index value and the cell index at the delta time point.

Additional migration assays were carried out in a 24‐well Transwell system (Corning) equipped with porous polycarbonate membranes (diameter 8 μm). The MSCs were seeded in Transwell inserts (10^4^ cells) and inserted into the lower chamber of the system 6 h after plating into the upper chamber. Myoblast‐conditioned medium, myoblast medium containing 2% FBS or regular medium with 10% FBS (positive control) were placed in the lower chamber. The system was kept at 5% CO_2_ for 24 h and the cells were then washed with PBS and fixed with 4% PFA. The cells from the inner surface of the membrane were removed with cotton buds. The membrane was then cut out, the cells on the underside of the membrane were stained with DAPI (Sigma) and analyzed using a KEYENCE BZ‐9000 fluorescence microscope (BIOREVO). Nuclei counting was performed in no less than five fields of view.

To analyze the mechanism of MSC migration, the media conditioned by normal and FSHD myoblasts were preincubated with 10 ng/ml neutralizing antibodies against CXCL12 (Abcam) for 30 min before migration analysis, or the MSCs were treated with 10 mM of synthetic CXCR4 inhibitor AMD3100 (Sigma) during the analysis.

### 2.10 Statistical analysis

Data are presented as means ± SD, the *p* values are as follows: *p < 0.05, **p < 0.01, ***p < 0.001, ****p < 0.0001. Student's two tailed *t*‐test or nonparametric Mann−Whitney test were used for pairwise comparisons depending on the data distribution. For multiple comparisons Kruskall−Wallis test with the false discovery rate (FDR) correction was performed. The statistics were calculated in GraphPad Prism 6 software.

## 3 RESULTS

Primary and IM isolated from FSHD patients have a similar doubling time but exhibit differentiation defects (Barro et al., 2010; Vilquin et al., 2005). We first compared the efficiency of myogenic differentiation of PM and IM myoblasts from healthy and FSHD donors used in this study. IM were derived from a healthy individual (AB1190) and from a patient with the FSHD (AB1080) (Mamchaoui et al., 2011) and immortalized as described elsewhere (Zhu et al., 2007). PM were isolated from skeletal muscle biopsies of healthy and FSHD subjects (Barro et al., 2010). In accordance with the previously published data (Barro et al., 2010; Vilquin et al., 2005), PMs and IMs derived from FSHD patients exhibited morphological differentiation defects: the diameter of myotubes formed by the myoblasts from FSHD patients was 1.5−2 times less than that of the control myoblasts (Supporting Information: Figure 1).

### 3.1 FSHD myoblasts stimulate MSC migration via CXCL12‐CXCR4 axis and enhance MSC proliferation

**Figure 1:**
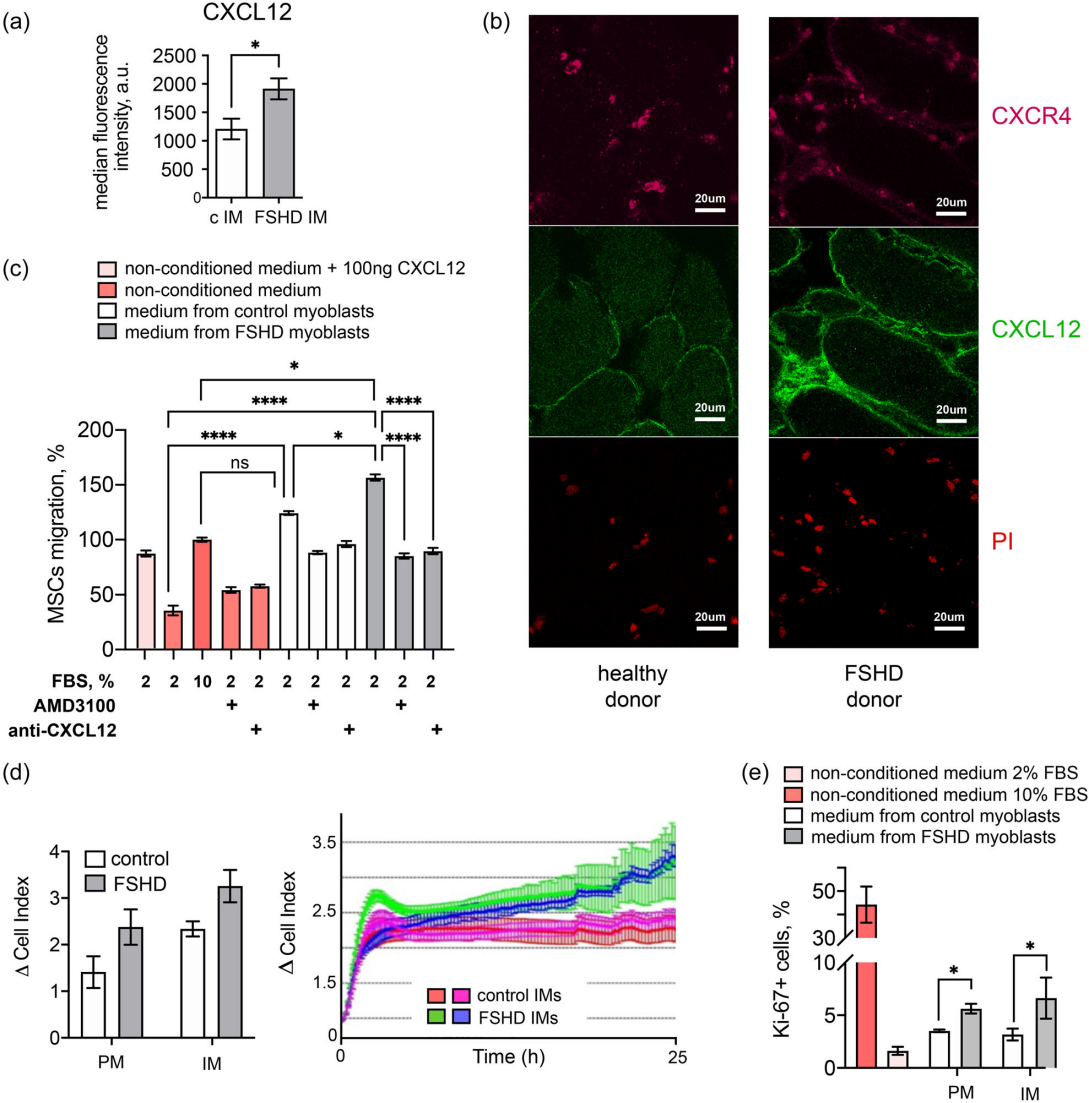
CXCL12 secreted from immortalized myoblasts stimulates MSCs migration and proliferation. (a) CXCL12 protein level in myoblasts detected by flow cytometry (mean ± SD, *p < 0.05, *n* = 7). (b) Representative image of immunohistochemical staining for CXCR4 (magenta) and CXCL12 (green), muscle biopsies obtained from an FSHD patient and a healthy donor; nuclei are stained with (p red). (c) MSCs migration to myoblast‐conditioned media supplemented with inhibitory factors. Delta cell index value of MSCs migration toward control, nonconditioned medium with 10% FBS is taken as 100% (mean ± SD, *p < 0.05, ****p < 0.0001, *n* = 5). (d) MSCs migration to myoblast‐conditioned media, delta cell index values at 23 h (left) and dynamic delta cell index values (right). (e) MSCs proliferation in conditioned and nonconditioned media assessed by the percentage of Ki‐67‐positive nuclei, flow cytometry data (mean ± SD, *p < 0.05, *n* = 3). FSHD, facioscapulohumeral muscular dystrophy; MSC, mesenchymal stem cells; propidium iodide (PI) PM, primary myoblasts.

The CXCL12‐CXCR4 chemokine signaling axis is involved in inflammation and promotes cell migration (reviewed in García‐Cuesta et al., 2019), including migration of MSCs (Hu et al., 2013). Abnormal MSC migration may be associated with fibrosis and fat replacement of muscle tissue (reviewed in (Guillamat‐Prats, 2021)), frequently observed in FSHD (Dahlqvist et al., 2019; Stadler et al., 2011). We have previously shown that *CXCL12* and *CXCR4* are overexpressed in *DUX4*‐expressing immortalized human myoblasts, leading to increased MSCs migration toward *DUX4*‐expressing IM (Dmitriev, Kiseleva et al., 2016). Using flow cytometry, we compared levels of CXCL12 protein in FSHD and the control IM. Immortalized FHSD myoblasts had a significantly higher CXCL12 level (1913 ± 487 v*s*. 1206 ± 513 a.u. for FSHD and normal myoblasts, respectively Figure 1a). A similar increase in the CXCL12 level was observed in a muscle biopsy from a FSHD patient, where CXCL12 accumulated in the interstitial regions (Figure 1b).

MSCs are attracted by CXCL12 to migrate toward the focus of inflammation (Marquez‐Curtis & Janowska‐Wieczorek, 2013). We next analyzed whether FSHD myoblasts could attract MSCs through the CXCL12‐CXCR4 axis by analyzing the real‐time MSC mobility towards the medium conditioned by either control or FSHD myoblasts. Indeed, the medium conditioned by PM and IM from FSHD patients stimulated MSCs migration 1.4 and 1.7‐fold compared to the medium conditioned by the control myoblasts (3.3 ± 0.3 vs. 2.3 ± 0.2 for IM and 2.4 ± 0.4 vs. 1.4 ± 0.3 for PM) (Figure 1d). To confirm that the enhanced migration of MSCs was due to an increased CXCL12 concentration in the FSHD myoblast‐conditioned media, we used neutralizing antibodies against CXCL12 and AMD3100, a synthetic antagonist of the CXCL12 receptor CXCR4. Preincubation of the medium conditioned by the normal and FSHD IM with neutralizing antibodies against CXCL12 or MSCs treatment with AMD3100 significantly reduced MSC migration (Figure 1c). As MSC mobility can also be regulated via CCL21‐CCR7 and CCL5‐CCR1,3,5 axes and CCL5 upregulation was detected in FSHD muscle biopsies (Rahimov et al., 2012), we also verified the expression of these chemokines and their receptors in normal, FSHD IM and in MSC cells. Though their expression was detected, the Ct‐s in myoblasts were very high (data not shown). The expression of CCL5 was elevated in FSHD IM thus CCL5/CCR5 signaling might contribute to MSC homing, but taking into account the overall low expression of CCL5 in FSHD IM and CCR5 in MSCs, this axis only plays an additional role to the CXCL12/CXCR4.

We also studied the effect of factors secreted by primary or IM from healthy and FSHD individuals on MSC proliferation. MSCs were serum‐starved for 48 h and then transferred to either myoblast‐conditioned medium or 10% FBS‐containing medium (positive control). 24 h post‐transfer, the number of proliferating Ki‐67 positive cells was analyzed by flow cytometry. The number of Ki‐67‐positive MSCs was ~1.5‐fold higher when the cells were exposed to the conditioned medium from FSHD myoblasts as compared to the conditioned medium from control ones (Figure 1e). Thus, myoblasts from FSHD patients enhance both MSCs migration through the CXCL12 axis and MSC proliferation.

### 3.2 FSHD myoblasts stimulate collagen synthesis and secretion in MSCs under inflammatory conditions

MSCs can produce and secrete extracellular matrix proteins, such as collagen and fibronectin, thus participating in the accumulation of extracellular matrix components and in fibrosis, a process of replacement of muscle tissue with the connective one (Mann et al., 2011). Increased collagen secretion by MSCs in the FSHD context may contribute to fibrosis development.

**Figure 2:**
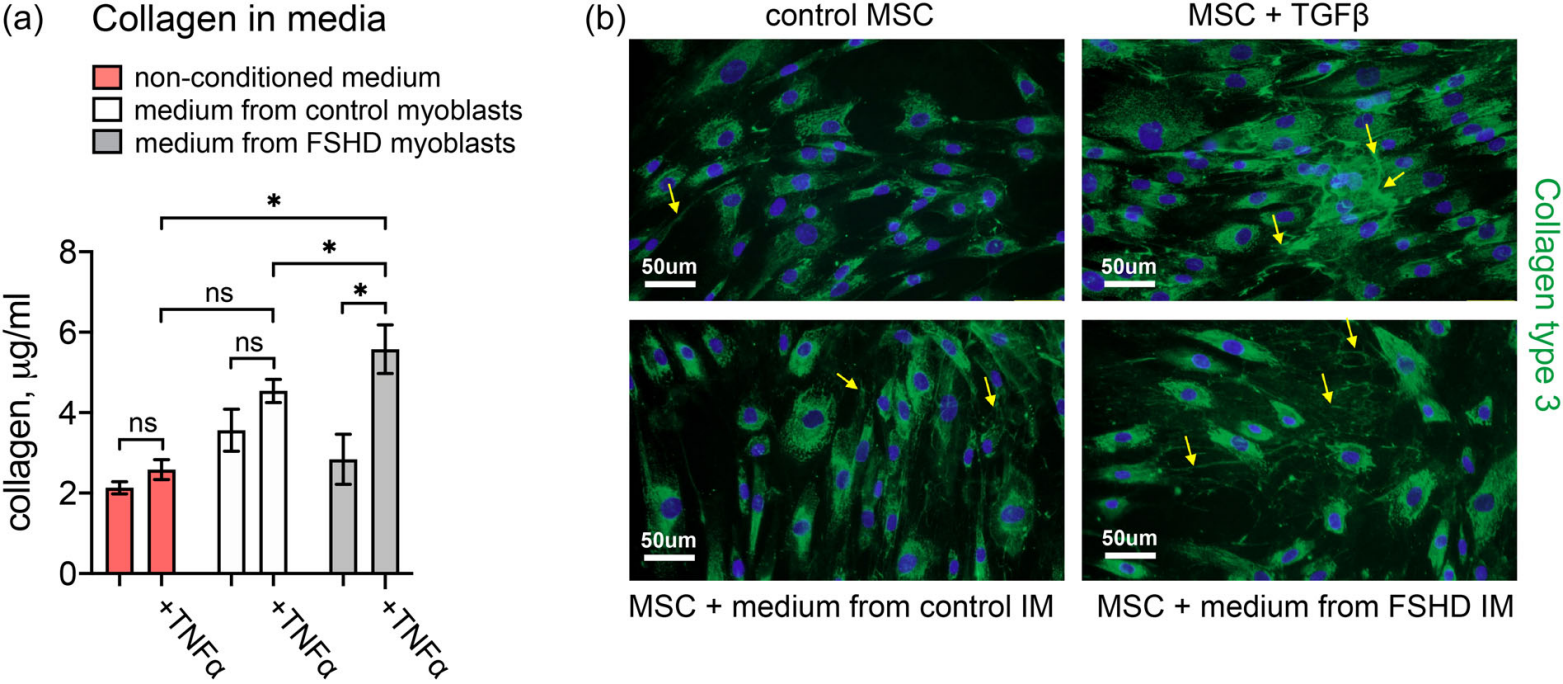
Collagen synthesis and secretion in MSCs cultivated in the media conditioned by control and FSHD myoblasts under regular and inflammatory conditions. (a) The cells were cultured in the conditioned or nonconditioned medium for 5 days, treated with TNF‐α for 24 h and then collagen concentrations in the MSC medium were measured; mean ± SD, *p < 0.05, *n* ≥ 3. (b) Representative images of immunohistochemical staining for collagen type III (green) and cell nuclei (blue) in MSCs treated as in (a). Scale bar: 50 um. FSHD, Facioscapulohumeral muscular dystrophy; MSC, mesenchymal stem cells.

We have assessed collagen secretion into the medium by the control MSCs and MSCs incubated in the media conditioned by the control and FSHD myoblasts using a method of collagen quantification based on the ability of Sirius Red dye to bind to fibrillar type I and III collagens. Collagen concentrations in the media were measured after 5 days of incubation in the conditioned media. The myoblast‐conditioned medium did not significantly affect collagen secretion under regular conditions (Figure 2a). MSCs are generally attracted by CXCL12 to migrate toward the focus of inflammation (Rustad & Gurtner, 2012). Inflammation induces loss of skeletal muscles and fibrosis (Londhe & Guttridge, 2015) and inflammation is characteristic of FSHD muscles (Arahata et al., 1995; Frisullo et al., 2011; Tasca et al., 2012). Thus, we next assessed collagen secretion under inflammatory conditions induced by TNF‐α. Upon TNF‐α treatment, collagen secretion into the medium increased, and the effect was more pronounced in the MSCs conditioned with the medium from FSHD myoblasts (Figure 2a). To confirm our biochemical results, we analyzed the collagen content by collagen immunostaining. The MSCs were fixed 24 h after treatment with the myoblast conditioned media. This time point was chosen to identify a rapid response to the treatment of MSCs with myoblast conditioned media. In our immunofluorescence analysis, anti‐collagen I antibodies stained only intracellular collagen. Since collagen type III is a major player in inflammatory associated matrix formation and wound healing and it is also a marker of severity of liver and tumor fibrosis (Nissen et al., 2022; Sørensen et al., 2022), we used anti‐collagen III antibody, which provided both intracellular and extracellular staining (Figure 2b). Both TGFb‐treated MSCs (positive control for collagen secretion) and MSCs treated with the FSHD myoblast conditioned media produced extracellular collagen type III, in contrast to the control MSC cells (Figure 2b).

### 3.3 MSC presence impairs myoblast differentiation

**Figure 3:**
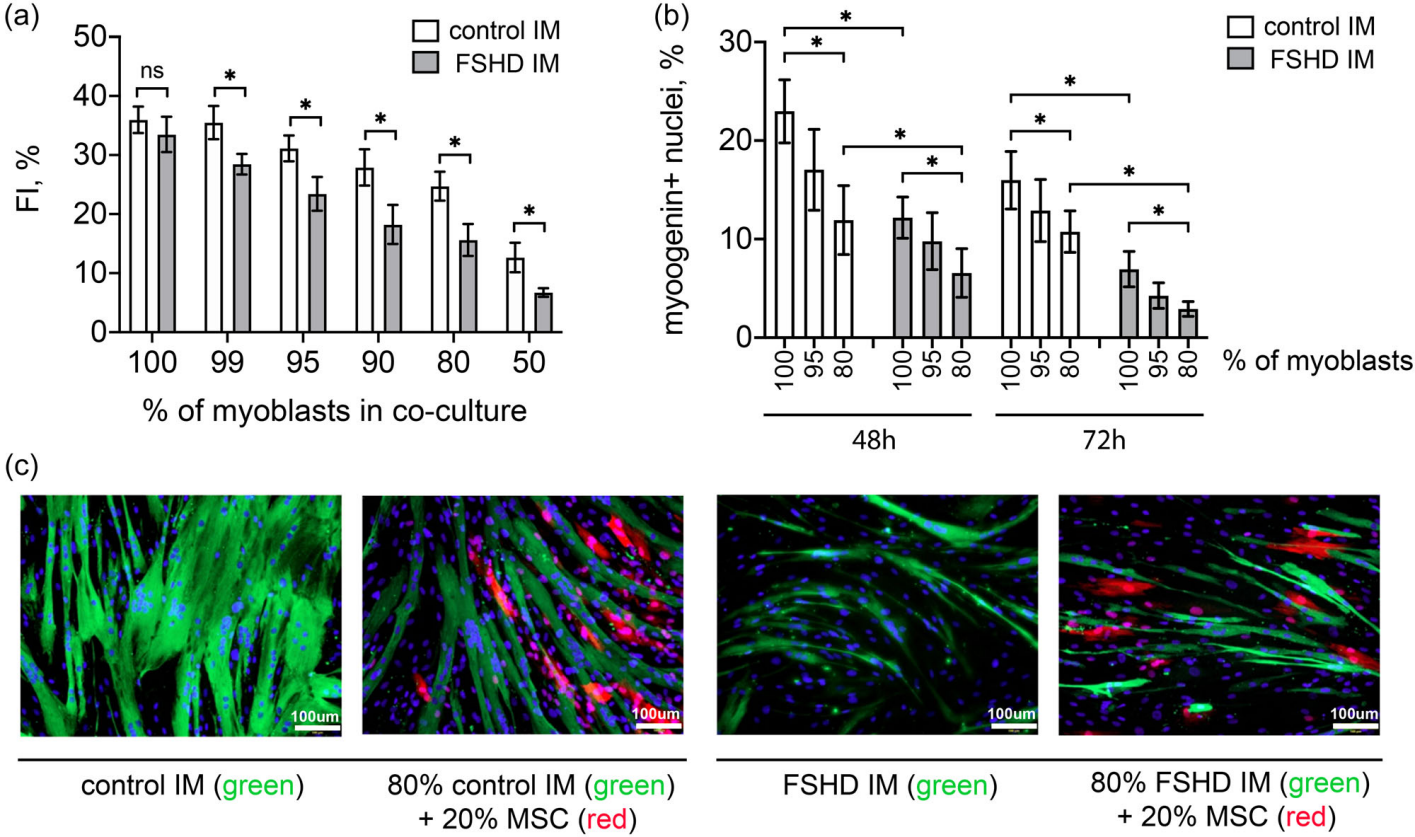
MSC presence impairs myogenic differentiation. (a) Fusion index of control and FSHD myoblasts cocultured with MSCs in different ratios 5 days after differentiation induction (mean ± SD, *p < 0.05, *n* ≥ 9). (b) Percentage of myogenin positive nuclei in IM‐MSC cocultures 48 and 72 h after differentiation induction. (c) Immunofluorescence staining in IM‐MSC cocultures 5 days after differentiation induction. Myotubes are visualized by MF20 staining (green), TaqRFP MSCs are red, nuclei are counterstained with DAPI (blue). Scale bar: 100 μm. FSHD, facioscapulohumeral muscular dystrophy; IM, immortalized myoblasts.

MSCs migrate to affected muscles and potentially contribute to fibrosis development, but their effect on the residing myoblasts remains unclear. In the presence of myoblasts, rat MSCs have a potential to differentiate into myotubes (Beier et al., 2011), therefore MSCs may affect myogenesis by direct fusion with the myoblasts. MSCs may also affect myogenesis indirectly via secreted paracrine factors. We cocultured healthy and FSHD IM with MSCs in different ratios (1%−50% MSCs), and induced myogenic differentiation after 5 days of cocultivation. The presence of any amount of MSCs decreased the FI; this effect was more pronounced in the FSHD myoblasts (Figure 3a). For example, the FI of FSHD myoblasts was reduced 1.8‐fold (18.3 ± 3.3 vs. 32.5 ± 3.0) in the coculture with 10% MSCs, while the FI of the control myoblasts in the same conditions was reduced only 1.3‐fold (27.9 ± 3.1 vs. 36.0 ± 2.2). The percentage of nuclei expressing the myogenic differentiation marker myogenin also decreased in the presence of MSCs proportionally to their percentage in the coculture (Figure 3b).

We then used the MSCs expressing red fluorescent protein (TaqRFP) and nonlabeled myoblasts and induced myogenic differentiation in the coculture for 5 days to detect hybrid myotubes potentially resulting from myoblast‐MSC fusion. Then the cultures were fixed and stained with myotubes marker MF20 antibody which recognizes the heavy chain of myosin II (green) (Figure 3c). In the coculture with MSCs, no hybrid myotubes were detected on the 5th day of differentiation in either condition (FSHD or control). Therefore, the presence of MSCs negatively affects myogenic differentiation and may contribute to muscle dysfunction in the FSHD context, but this effect is not mediated by direct MSC‐myoblast fusion in our system.

## 4 DISCUSSION

Skeletal muscle comprises a population of fibroadipogenic progenitors/MSCs in addition to myotubes and myogenic precursor (satellite) cells. In physiological conditions, MSCs participate in muscle regeneration (Joe et al., 2010; Wosczyna et al., 2019), while chronic inflammation or persistent muscle damage may induce MSCs to differentiate into adipocytes or contribute to muscle fibrosis (Uezumi et al., 2010). MSCs are found in many organs and tissues; their native niche is a perivascular space (Crisan et al., 2008) from where they can migrate following specific signals including the CXCL12‐CXCR4 signaling axis (Marquez‐Curtis & Janowska‐Wieczorek, 2013). The CXCL12‐CXCR4 signal axis controls organogenesis, hematopoiesis, angiogenesis, neurogenesis, migration of immune and primary germ cells, and the preservation of hematopoietic stem cells in the bone marrow (reviewed in Bianchi & Mezzapelle, 2020). Misregulation of the CXCR4‐CXCL12 signaling axis is associated with numerous pathological conditions, including various cancers, chronic inflammatory diseases, cardiovascular diseases, and immunodeficiencies (Britton et al., 2021).

FSHD is a muscular disorder strongly associated with an aberrant expression of the *DUX4* gene (Lemmers et al., 2010). In addition to progressive muscle weakness and wasting, FSHD histopathology includes fibrosis and muscle replacement with fat and connective tissues (Dahlqvist et al., 2019; DeSimone et al., 2020; Stadler et al., 2011). We have previously shown that myoblasts expressing *DUX4* overexpressed *CXCL12* and secreted CXCL12 both in vivo and in vitro (Dmitriev, Kiseleva et al., 2016; p. 4 and Figure 2), which may attract MSCs that express the CXCR4 receptor to the FSHD muscles. Indeed, MSC cells migrate more actively towards *DUX4*‐expressing myoblasts (Dmitriev, Kiseleva et al., 2016; p. 4), and the muscles of mice with stochastic low level *DUX4* expression exhibit a remarkable expansion in the fibroadipogenic progenitor compartment (Bosnakovski et al., 2017, 2020), indicating an increased MSC presence. What would be the functional consequences of the enhanced presence of MSCs?

Here we investigated the mutual effects of MSCs and myoblasts in a cocuture model of FSHD. We demonstrated that the media conditioned by myoblasts from FSHD patients induced MSC migration in a CXCL12‐dependent manner and stimulated MSC proliferation (Figure 1). Myoblast‐conditioned media was able to increase collagen secretion by MSCs, but only under inflammatory conditions (Figure 2). This is in line with what was previously observed both in vivo in murine models and in FSHD patients. In FSHD mouse models, muscle degeneration involved inflammation (Arahata et al., 1995; Bosnakovski et al., 2017, 2020; Choi et al., 2017; Frisullo et al., 2011; Tasca et al., 2012) and elevation of collagen deposition between the affected myofibers (Bosnakovski et al., 2020). MRI of FSHD muscles indicated an inflammatory process with lymphocyte invasion (Arahata et al., 1995; Frisullo et al., 2011; Tasca et al., 2012) and the progression of muscle replacement was higher in the inflamed compared to noninflamed FSHD muscles (Dahlqvist et al., 2019). Notably, CXCL12‐CXCR4 axis can modulate inflammatory response and be itself affected by inflammation (Lau et al., 2020). Pretreatment of bone marrow MSCs with TNF‐α enhances MSC migration. TNF‐α increases expression of CCR2, CCR3, CCR4, but not CXCR4 in MSCs (Ponte et al., 2007). In our work, the presence of TNF‐α alone in the culture medium did not induce MSC migration per se. TNF‐α may affect MSC sensitivity to CXCL12 by modulating CXCR4 signal transmission (Petit et al., 2005), but in our in vitro system, MSCs migrating towards myoblast‐conditioned media were not pretreated with TNF‐α. It is thus likely that inflammation would further increase MSC migration. Thus, MSCs may migrate to inflamed FSHD muscles due to increased CXCL12 production by FSHD myoblasts, and contribute to muscle fibrosis through increased collagen secretion. We also evaluated the effect of MSCs on myogenesis in the MSCs‐myoblasts cocultures. The presence of just 5% of MSCs impaired myoblasts fusion, and the effect was more pronounced for FSHD myoblasts (Figure 3). Therefore, MSC presence may impair myogenic differentiation and contribute to reduced muscle regeneration observed in FSHD. Can MSC presence in FSHD muscles be modulated? One possible way is to target the CXCL12‐CXCR4 axis. Indeed we have shown that treatment with neutralizing antibodies against CXCL12 or with CXCR4 inhibitor AMD3100 reduced MSC migration to the control levels. This approach will be tested in future in murine models of FSHD.

## AUTHOR CONTRIBUTIONS

Yegor S. Vassetzky and Ekaterina Kiseleva designed and conceptualized the study. Ekaterina Kiseleva, Olesia Serbina, and Anna Karpukhina performed experiments. Ekaterina Kiseleva, Olesia Serbina, and Anna Karpukhina analyzed data. Yegor S. Vassetzky, Ekaterina Kiseleva, Olesia Serbina, and Anna Karpukhina wrote the manuscript. All the authors revised and approved the manuscript's final version.

## CONFLICT OF INTEREST

The authors declare no conflict of interest.

## Supporting information

Supporting information.
