# Supplementary material for: Interaction between mesenchymal stem cells and myoblasts in the context of facioscapulohumeral muscular dystrophy contributes to the disease phenotype
Source: J Cell Physiol. 2022 May 27;237(8):3328–37. doi: 10.1002/jcp.30789 (PMC9545833; doi:10.1002/jcp.30789)
Supplement: Supplementary file 1 — Supporting information. [file JCP-237-3328-s001.pdf]

Supplementary data

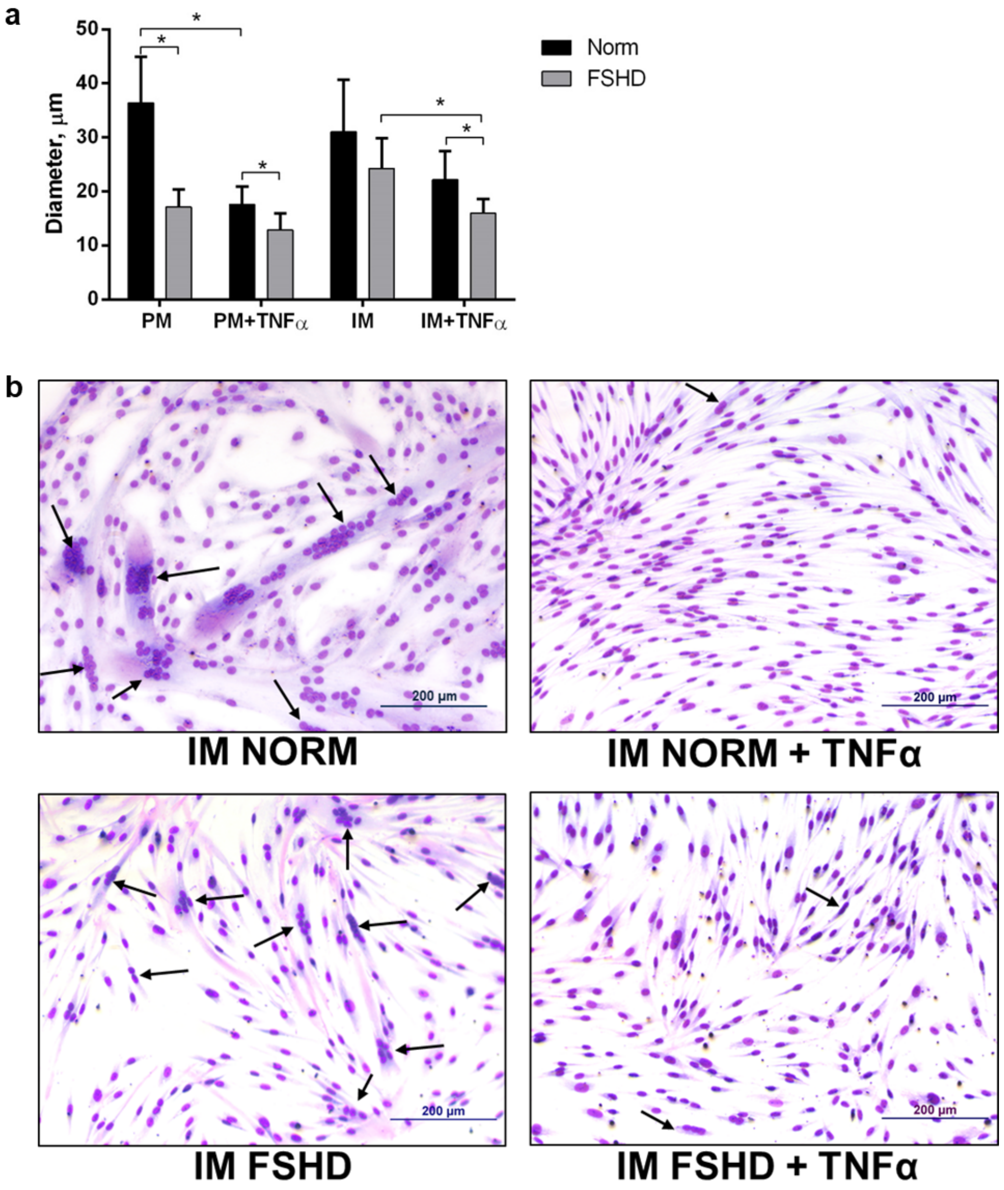

Supplementary figure 1. Differentiation of control and FSHD myoblasts in normal conditions and in the inflammatory conditions induced by TNFα. **(A)** Myotube diameters (mean ± SD, * - $p < 0.05$, $n \geq 5$). **(B)** Representative microscopic fields. Myotubes are marked with arrows. (Jenner-Giemsa staining, scale bar: 200 μm). IM - immortalized myoblasts.

Supplementary table 1. List of primary antibodies

**Name**	**Dilution**	**Manufacturer**	**Catalog number**
mouse monoclonal anti-myogenin	1:100	Abcam	ab1835
mouse monoclonal anti-Ki67	1:100	Sigma	P6834
mouse monoclonal anti-SDF1 (CXCL12)	1:50	Santa Cruz Biotechnology	SC-518066
mouse monoclonal anti-MF20	1:500	Thermo Fisher	14-6503-82
Rabbit monoclonal anti-CXCR4	1:500	Abcam	ab124824

Supplementary table 2. List of secondary antibodies

**Name**	**Manufacturer**	**Catalog number**
Alexa Fluor 488 donkey anti-mouse IgG (H+L) (2 mg/ml)	Life Technologies	A-21202
Alexa Fluor 488 goat anti-mouse IgG (H+L) (2 mg/ml)	Life Technologies	A-11029
Alexa Fluor 594 chicken anti-mouse IgG (H+L) (2 mg/ml)	Life Technologies	A-21201
Alexa Fluor 594 chicken anti-rabbit IgG (H+L) (2 mg/ml)	Life Technologies	A-21442
Alexa Fluor 594 donkey anti-mouse IgG (H+L) (2 mg/ml)	Life Technologies	A-21203
Alexa Fluor 594 donkey anti-rabbit IgG (H+L) (2 mg/ml)	Life Technologies	A-21207
